# Structure of Chimpanzee Gut Microbiomes across Tropical Africa

**DOI:** 10.1128/mSystems.01269-20

**Published:** 2021-06-22

**Authors:** Clifton P. Bueno de Mesquita, Lauren M. Nichols, Matthew J. Gebert, Caihong Vanderburgh, Gaëlle Bocksberger, Jack D. Lester, Ammie K. Kalan, Paula Dieguez, Maureen S. McCarthy, Anthony Agbor, Paula Álvarez Varona, Ayuk Emmanuel Ayimisin, Mattia Bessone, Rebecca Chancellor, Heather Cohen, Charlotte Coupland, Tobias Deschner, Villard Ebot Egbe, Annemarie Goedmakers, Anne-Céline Granjon, Cyril C. Grueter, Josephine Head, R. Adriana Hernandez-Aguilar, Kathryn J. Jeffery, Sorrel Jones, Parag Kadam, Michael Kaiser, Juan Lapuente, Bradley Larson, Sergio Marrocoli, David Morgan, Badru Mugerwa, Felix Mulindahabi, Emily Neil, Protais Niyigaba, Liliana Pacheco, Alex K. Piel, Martha M. Robbins, Aaron Rundus, Crickette M. Sanz, Lilah Sciaky, Douglas Sheil, Volker Sommer, Fiona A. Stewart, Els Ton, Joost van Schijndel, Virginie Vergnes, Erin G. Wessling, Roman M. Wittig, Yisa Ginath Yuh, Kyle Yurkiw, Klaus Zuberbühler, Jan F. Gogarten, Anna Heintz-Buschart, Alexandra N. Muellner-Riehl, Christophe Boesch, Hjalmar S. Kühl, Noah Fierer, Mimi Arandjelovic, Robert R. Dunn

**Affiliations:** a Department of Ecology and Evolutionary Biology, University of Colorado, Boulder, Colorado, USA; b Cooperative Institute for Research in Environmental Sciences, University of Colorado, Boulder, Colorado, USA; c Department of Applied Ecology, North Carolina State University, Raleigh, North Carolina, USA; d Max Planck Institute for Evolutionary Anthropology, Leipzig, Germany; e Jane Goodall Institute Spain and Sénégal, Dindefelo Biological Station, Dindefelo, Kedougou, Sénégal; f School of Biological & Environmental Sciences, Liverpool John Moores University, Liverpool, United Kingdom; g Department of Psychology, West Chester University, West Chester, Pennsylvania, USA; h Department of Anthropology & Sociology, West Chester University, West Chester, Pennsylvania, USA; i Chimbo Foundation, Oudemirdum, The Netherlands; j School of Human Sciences, University of Western Australia, Perth, Australia; k School of Biological Sciences, University of Western Australia, Perth, Australia; l International Centre of Biodiversity and Primate Conservation, Dali University, Dali, Yunnan, China; m Department of Social Psychology and Quantitative Psychology, Faculty of Psychology, University of Barcelona, Barcelona, Spain; n School of Natural Sciences, University of Stirling, Stirling, United Kingdom; o Royal Society for the Protection of Birds, Cambridge, United Kingdom; p Greater Mahale Ecosystem Research and Conservation Project, Tanzania; q Comoé Chimpanzee Conservation Project, Kakpin, Comoé National Park, Ivory Coast; r Lester E. Fisher Center for the Study and Conservation of Apes, Lincoln Park Zoo, Chicago, Illinois, USA; s Leibniz Institute for Zoo and Wildlife Research, Berlin, Germany; t Institute of Tropical Forest Conservation (ITFC), Mbarara University of Science and Technology (MUST), Kabale, Uganda; u Wildlife Conservation Society, Bronx, New York, USA; v Projet GALF-Guinée, Wara Conservation Project, Guinea; w Department of Anthropology, University College London, London, United Kingdom; x Department of Anthropology, Washington University in Saint Louis, St. Louis, Missouri, USA; y Forest Ecology and Forest Management Group, Wageningen University & Research, Wageningen, The Netherlands; z Gashaka Primate Project, Serti, Nigeria; aa Wild Chimpanzee Foundation, Leipzig, Germany; bb Department of Human Evolutionary Biology, Harvard University, Cambridge, Massachusetts, USA; cc Taï Chimpanzee Project, CSRS, Abidjan, Ivory Coast; dd Department of Geography, Planning and Environmental Studies, University of Concordia, Montréal, Quebec, Canada; ee Institut de Biologie, Université de Neuchâtel, Neuchâtel, Switzerland; ff School of Psychology and Neuroscience, University of St. Andrews, St. Andrews, United Kingdom; gg Epidemiology of Highly Pathogenic Microorganisms, Robert Koch Institute, Berlin, Germany; hh Viral Evolution Project Group, Robert Koch Institute, Berlin, Germany; ii German Centre for Integrative Biodiversity Research (iDiv) Halle-Jena-Leipzig, Leipzig, Germany; jj Department of Soil Ecology, Helmholtz Centre for Environmental Research GmbH - UFZ, Halle, Germany; kk Department of Molecular Evolution and Plant Systematics & Herbarium (LZ), Institute of Biology, Leipzig University, Leipzig, Germany; University of Connecticut

**Keywords:** prokaryotes, parasites, diet, tools, host genetics, climate

## Abstract

Understanding variation in host-associated microbial communities is important given the relevance of microbiomes to host physiology and health. Using 560 fecal samples collected from wild chimpanzees (Pan troglodytes) across their range, we assessed how geography, genetics, climate, vegetation, and diet relate to gut microbial community structure (prokaryotes, eukaryotic parasites) at multiple spatial scales. We observed a high degree of regional specificity in the microbiome composition, which was associated with host genetics, available plant foods, and potentially with cultural differences in tool use, which affect diet. Genetic differences drove community composition at large scales, while vegetation and potentially tool use drove within-region differences, likely due to their influence on diet. Unlike industrialized human populations in the United States, where regional differences in the gut microbiome are undetectable, chimpanzee gut microbiomes are far more variable across space, suggesting that technological developments have decoupled humans from their local environments, obscuring regional differences that could have been important during human evolution.

**IMPORTANCE** Gut microbial communities are drivers of primate physiology and health, but the factors that influence the gut microbiome in wild primate populations remain largely undetermined. We report data from a continent-wide survey of wild chimpanzee gut microbiota and highlight the effects of genetics, vegetation, and potentially even tool use at different spatial scales on the chimpanzee gut microbiome, including bacteria, archaea, and eukaryotic parasites. Microbial community dissimilarity was strongly correlated with chimpanzee population genetic dissimilarity, and vegetation composition and consumption of algae, honey, nuts, and termites were potentially associated with additional divergence in microbial communities between sampling sites. Our results suggest that host genetics, geography, and climate play a far stronger role in structuring the gut microbiome in chimpanzees than in humans.

## INTRODUCTION

Explaining differences among individuals and species in microbiome composition, including gut microbiomes, has become a key dimension of research into host-microbe interactions and the effects of changes in microbiome on host health and physiology. Such differences have the potential to influence metabolic functions, nutrition, disease resistance, and cognitive performance (1, 2). One challenging feature of this work, particularly in humans and other species in which behavior differs among populations, has been to understand the relative importance of dietary choices, environment, geography, and host genetics in structuring microbiomes. We consider these factors in light of a species-wide study of the gut microbiomes, including bacteria, archaea, and eukaryotic parasites, of wild chimpanzees (Pan troglodytes) from 29 sites in Africa ranging from 50 km to 5,130 km apart.

Perhaps the simplest explanation for variation in gut microbiome communities among individuals within a species is a model in which infants acquire microbiomes from their mothers (3, 4) and then have those microbiomes supplemented and modified via interactions with their social groups as a function of interaction frequency (5, 6) and interactions with their environment (e.g., microbial exposures in soil, food, and water) (7). This explanation predicts that microbiomes diverge among populations and lineages as a function of genetic and geographic isolation and the consequent lack of contact and opportunities for microbial transmission. Such a model reflects processes that are neutral (or indistinguishable from neutral) with regard to local adaptation. In such a model, differences in host neutral genetic divergence should be highly predictive of differences in microbiome composition. However, microbiomes might be expected to “record” differences among populations in terms of their isolation with more nuance than is seen as a function of neutral genes of hosts alone, whether due to rapid host-dependent evolutionary changes (8, 9) or changes in which different microbial taxa are extirpated from or colonize hosts (10).

Additional drivers might be expected to accelerate microbiome divergence among populations. Two related factors suggested to have strong effects on microbiomes are climate and habitat, where habitat type and specific vegetation composition are influenced by climate but also by local factors such as soil type, slope, and land use (11). It is unlikely that climate and vegetation have a direct effect on gut microbiota; few microbes in mammalian guts have life histories that allow them to survive and proliferate outside the gut, with some parasites as a notable exception. However, climate and habitat determine the foods that are available to hosts (12), the abundance and behavior of vectors for parasites (13), and the immunological condition of the hosts (14). As a result, individuals that live in different climates and habitats, with different plant species composition, are expected to have more distinct gut microbiomes, especially with respect to parasites, than would be expected based on a neutral model alone. Interestingly, humans are an exception to this case, as the important factors of diet and lifestyle can be decoupled from climate and geography, especially in industrialized societies. For example, there can be major differences in gut microbiota composition between human populations with different cultural traditions and diets, but minor differences among regions within the same cultural tradition and diet (15).

**FIG 1:**
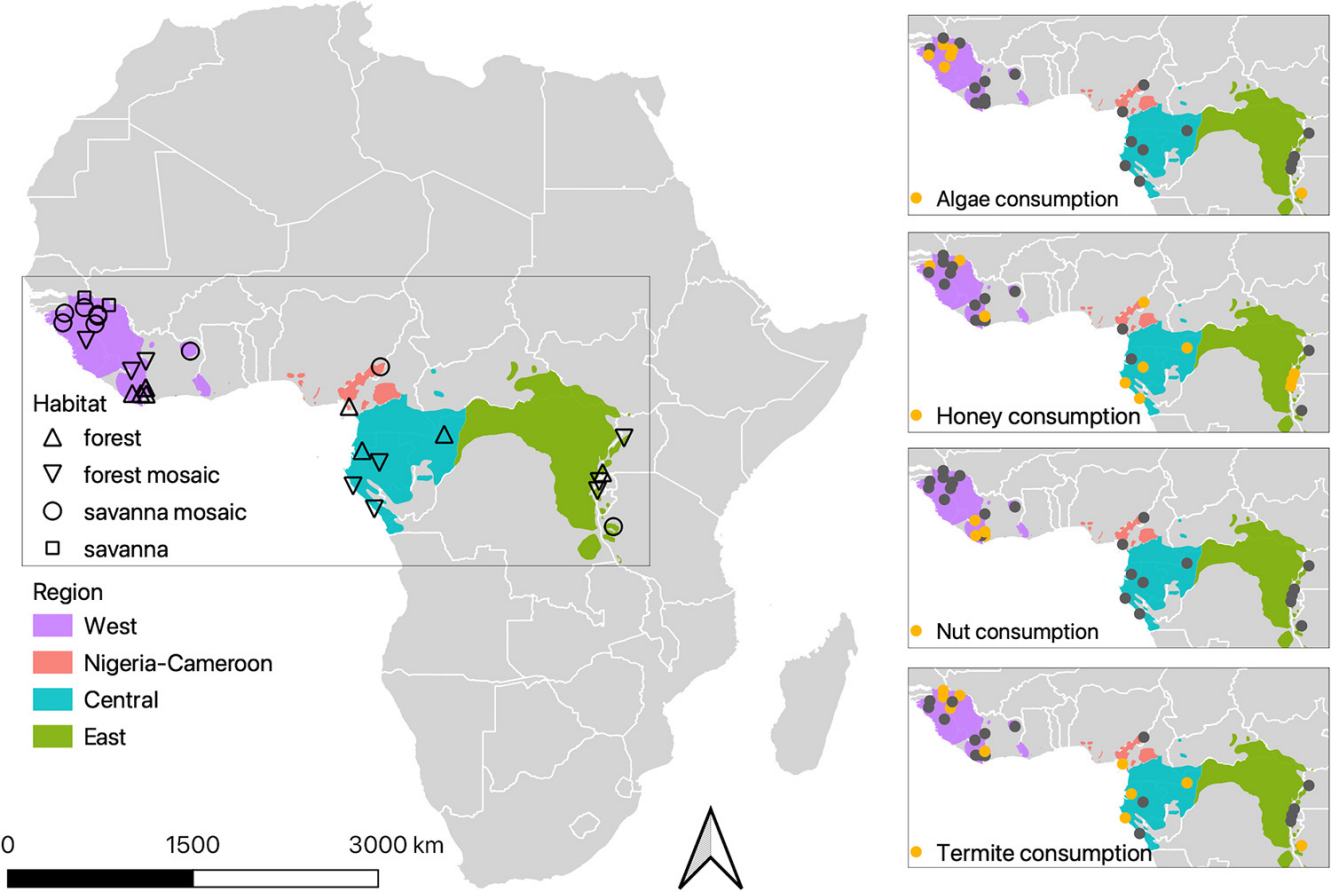
Map (from https://open.africa/) of the 29 sites included in this study, showing either forest, forest mosaic, savanna mosaic, or savanna habitat types and the ranges of the four main geographic regions of chimpanzees (from reference 133). Insets show the variation among sites (points) in consumption of algae, honey, nuts (hard-shelled drupes), and termites (orange = consumed, gray = not consumed). In many cases, these items are accessed using tools (algae [6 of 7 sites], honey [10 of 13 sites], nuts [5 of 5 sites], termites [9 of 12 sites]).

In addition to the indirect effects of climate and habitat on microbiomes via diet and host effects, there might also be effects of dietary choice on microbiomes (16). This is the case in humans, where individuals living in the same region may have different diets and, consequently, different microbiomes (1, 17, 18). A corollary of this pattern is apparent in evolutionarily distinct species that share diets and have similar gut microbiomes (19). Four chimpanzee subspecies are currently recognized (20) and inhabit a range of ecotones from dense forest to forest mosaics to savannas across equatorial Africa (Fig. 1) (21). They are considered frugivorous omnivores across their range, although forest-dwelling populations exhibit more dietary variety and frugivory than their savanna-woodland counterparts (22–24). The degree of faunivory (including insectivory) is also highly variable across the range (25, 26). However, even in similar and/or nearby environments, chimpanzees can have different diets (12, 27). This appears to be particularly common where nearby chimpanzee communities differ in their use of tools (28–33). For example, chimpanzees in Gombe National Park, Tanzania, forage on army ants (*Dorylus* spp.) and acrobat ants (*Crematogaster* spp.) using stick tools (32). In contrast, chimpanzees in Mahale Mountains National Park, less than 140 km from Gombe and with a similar habitat type in which army ants are common, do not forage on army ants but rather on carpenter ants (*Camponotus* spp.) (34). Similar differences are noted among chimpanzee communities in similar habitats with regard to the consumption of fruits and leaves (35), termites (31), honey (36), algae (30, 37), and some nut species that require tools to crack (29). Such behavioral differences in foraging techniques, presumed to be socially mediated and thus cultural (38), and consequent differences in diet, may in turn affect gut microbiota composition. For example, the use of tools facilitates access to dietary items that are relatively hard to digest, such as algae (those with stronger cellulose or silica cell walls), and items such as nuts that have unique protein and fat profiles relative to a predominantly fruit- and leaf-based diet.

Until very recently, the idea of comparing the microbiome of chimpanzees across populations spanning the full extent of their extant geographic range was unfeasible. Recently, however, the “Pan African Programme: The Cultured Chimpanzee” (PanAf; http://panafrican.eva.mpg.de/) has studied the behavioral diversity of chimpanzees across Africa (39) and sampled chimpanzee feces across that same geographic range (40). Building on these data and samples, it is possible to disentangle the relative influence of host genetics (and evolutionary history), climate, vegetation composition, and tool use on chimpanzee microbiomes, including both prokaryotes (bacteria and archaea) as well as eukaryotic parasites (including protist and nematode parasites and note that we use a lenient definition of parasites to include organisms that may not be harmful to the host or were originally listed as parasites but are now known to operate on a spectrum). Lastly, as chimpanzees are among the most closely related extant species to humans and comparisons are useful for understanding the evolution of the human gut microbiome (41), we compared the effects of geography, climate, and sex on the gut microbiomes of humans and chimpanzees. We hypothesized the following. (i) Large-scale geographic, genetic, and habitat differences among sites would drive differences in gut microbial community structure at the continental scale. (ii) Within regions, differences in diet due to vegetation composition and tool use would cause divergence in gut microbial community structure even within genetically similar populations owing to environmental and potential cultural influences on diet. (iii) Variation in the composition of gut parasite communities would mirror the observed variation in the prokaryotic communities due to similar factors influencing both components of the gut microbiome.

## RESULTS

DNA was extracted from 560 fecal samples (originating from 560 different individuals) collected across the chimpanzee geographic range (Fig. 1), and we used 16S and 18S rRNA marker gene sequence data to characterize the prokaryotic (bacteria and archaea) communities and eukaryotic (parasite) communities, respectively, in each fecal sample. Permutational multivariate analysis of variance (PERMANOVA) and Mantel tests were conducted on pairwise Bray-Curtis dissimilarities (prokaryotes) and Jaccard dissimilarities (parasites) to test for the effects of genetics, geography, climate, habitat, diet, and sex (see Materials and Methods).

**FIG 2:**
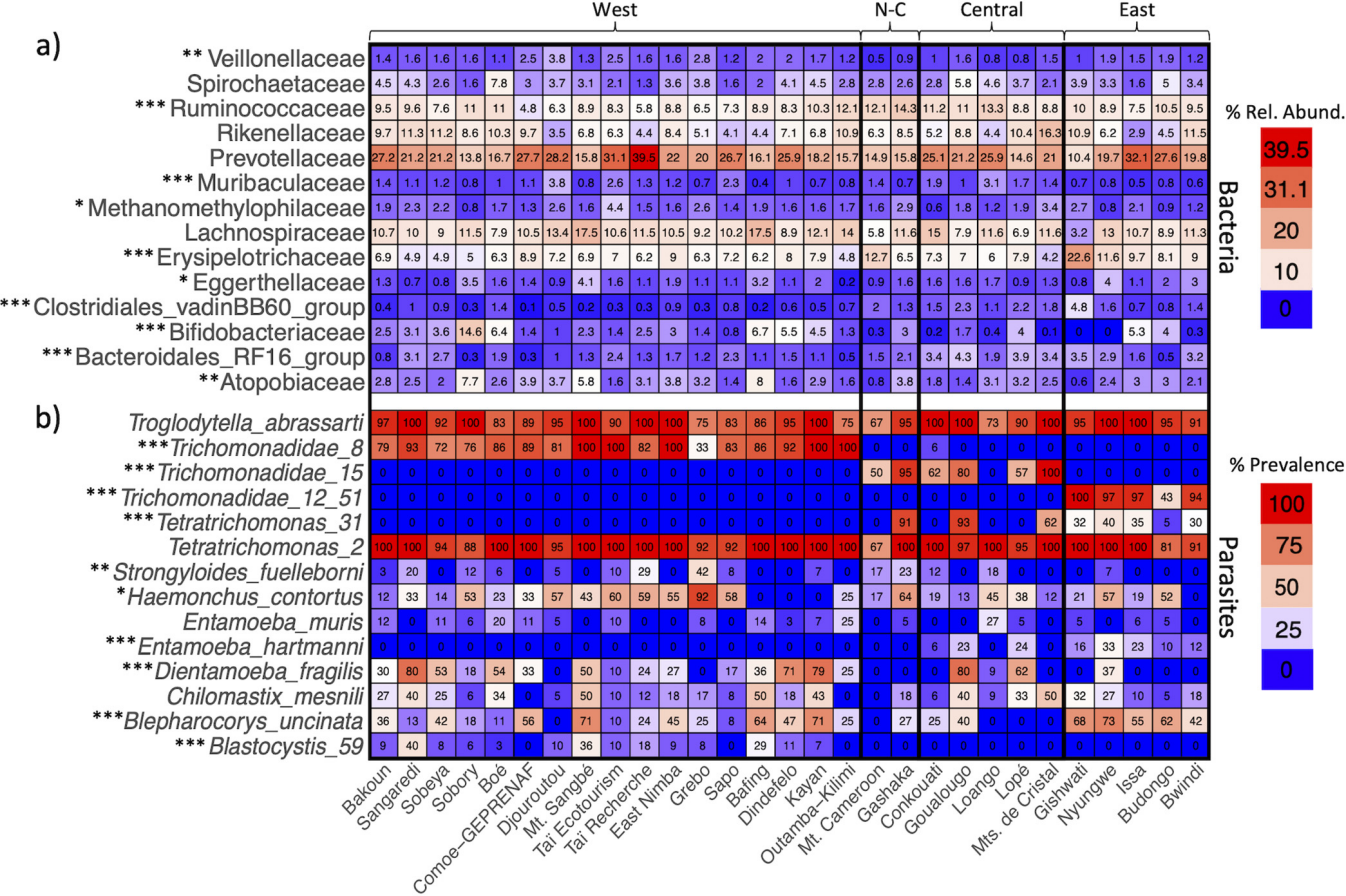
Heatmaps showing site mean percent relative abundances for 14 bacterial families with >1% mean relative abundance in at least one site (a) and parasite percent prevalence at each site (number of samples with parasite/total samples at site × 100) (b). Each panel is sorted from top to bottom in order of abundance (a) or prevalence (b). Note that there were 225 prokaryote families in the data set and if they were all included, columns would sum to 100. Different numbers after genera or families denote different ASVs; Trichomonadidae_12_51 represents two highly correlated ASVs that were combined. For more information about parasite taxonomy, see Table S3 at https://doi.org/10.6084/m9.figshare.14390426. Asterisks in panel a denote taxa with significantly different mean relative abundances among regions (Kruskal-Wallis, Bonferroni, *, *P* < 0.05; **, *P* < 0.01; ***, *P* < 0.001), and asterisks in panel b indicate taxa with significantly different probabilities of occurrence among regions (logistic regression, *, *P* < 0.05; **, *P* < 0.01; ***, *P* < 0.001). N-C, Nigeria-Cameroon.

There were 14 bacterial families with mean relative abundances (number of reads out of 8,000 reads per sample) greater than 1% in at least one site. Among these, 10 families had significantly different relative abundances among the four regions (Kruskal-Wallis, Bonferroni, *P* < 0.05, Fig. 2a). The most abundant bacterial family was *Prevotellaceae* (mean ± standard error [SE] percent abundance, 22.1 ± 0.5), followed by *Lachnospiraceae* (10.5 ± 0.3), *Ruminococcaceae* (9.3 ± 0.2), *Erysipelotrichaceae* (7.9 ± 0.2), and *Rikenellaceae* (7.9 ± 0.3), all of which are the typical dominant taxa seen in other studies of chimpanzees, humans, and other primates (18, 42–44). None of the most common taxa were likely to be associated with soil, leaves, or other potential field contaminants (45, 46). Among the 14 parasite taxa examined here, 10 had significantly different occurrence probabilities among regions (logistic regression, *P* < 0.05, Fig. 2b). The two most prevalent parasites across the whole data set, and the only two parasites present in all sites, were identified as Troglodytella abrassarti and a *Tetratrichomonas* amplicon sequence variant (ASV) most closely related to Tetratrichomonas buttreyi. For the other parasites, there were large differences in prevalence among the regions, with some regions having no occurrences of a particular parasite and other regions having almost 100% prevalence.

**FIG 3:**
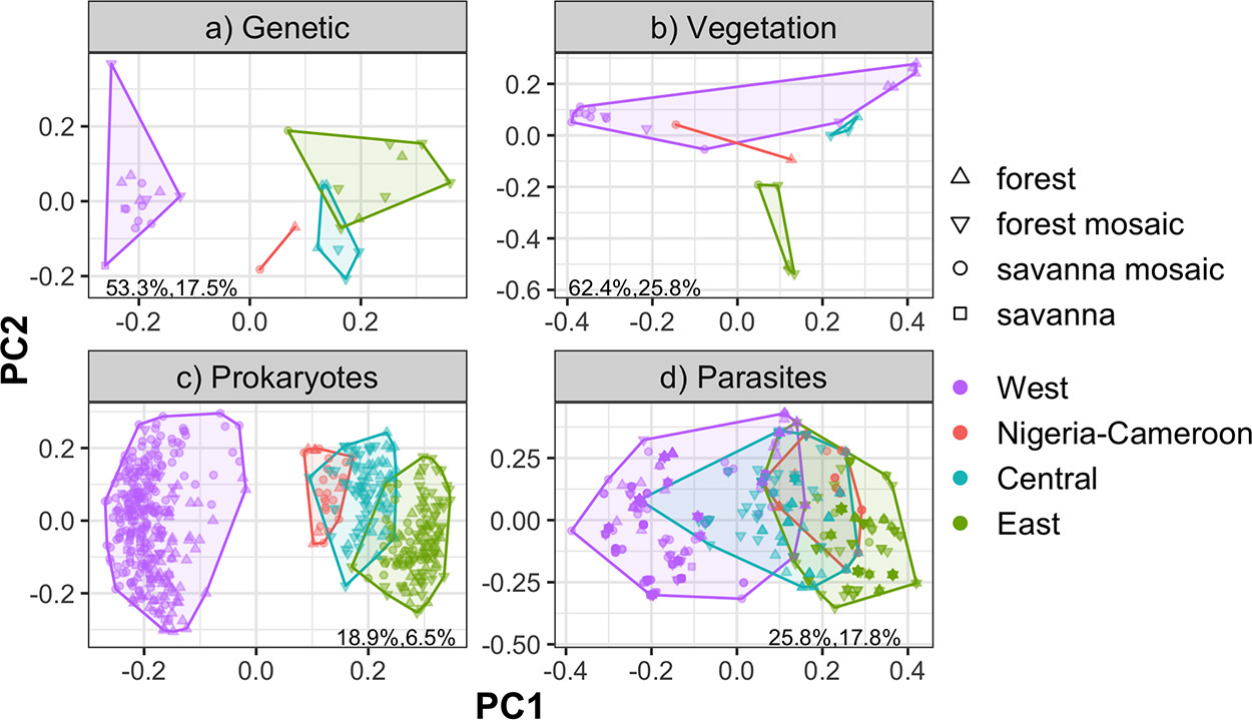
Principal coordinate analysis of chimpanzee site-level genetic distance [*F*′_ST_/(1 − *F*′_ST_)] (a), site-level vegetation distance (Bray-Curtis) (b), prokaryote (bacteria and archaea) community dissimilarity (Bray-Curtis) (c), and ubiquitous parasite community dissimilarity (Jaccard) (d). Numbers in the bottom corners represent the percent variation explained by principal coordinate 1 (PC1) and PC2, respectively. Point colors and convex hulls delineate the four geographic regions. *n* = 32, 27, 560, and 560 for a, b, c, and d, respectively. Overall, much of the structure in microbiomes is accounted for by genetic distance; note that the clustering in panel c matches the clustering in panel a rather than panel b. To see climate distances by region, see Fig. S2 at https://doi.org/10.6084/m9.figshare.14607735.

Geographic distance was significantly correlated with genetic, climate, and vegetation distance across the whole data set and within some, but not all, regions (Mantel test, *P* = 0.001, see Fig. S1 at https://doi.org/10.6084/m9.figshare.14607732). Geographic regions contained significantly different genetic populations of chimpanzees (PERMANOVA, pseudo-*F *= 15.6, *R*^2^ = 0.63, *P* = 0.001, Fig. 3a) as has been described elsewhere (47). Vegetation composition varied significantly among regions (PERMANOVA, *F *= 2.3, *R*^2^ = 0.23, *P* = 0.001, Fig. 3b). Regions also contained significantly different climates (PERMANOVA, pseudo-*F *= 4.5, *R*^2^ = 0.35, *P* = 0.001, see Fig. S2a at https://doi.org/10.6084/m9.figshare.14607735). Visual principal coordinate analysis (PCoA) grouping of samples by both vegetation composition and climate did not match the host genetic clustering, with the Nigeria-Cameroon, Central, and East regions grouped genetically (Fig. 3a), and the Nigeria-Cameroon, Central, and West regions grouped by vegetation (Fig. 3b) and climate (Fig. S2a at the figshare URL above).

### Variation in gut microbiota among regions.

Prokaryotic community composition differed significantly among the four geographic regions (Table 1 and Fig. 3c), with differences in the fecal microbial communities well correlated with site-level and individual-level genetic differences (Fig. 3a and c; see also Fig. S3 at https://doi.org/10.6084/m9.figshare.14607738). Parasite community composition also varied significantly among the four geographic regions (Table 1 and Fig. 3d). For both prokaryotes and parasites, all pairwise comparisons between regions were significant (*P* < 0.05). Additionally, parasite and prokaryotic community dissimilarities were significantly correlated across the whole data set (Mantel test, *P* = 0.001, see Fig. S4 at https://doi.org/10.6084/m9.figshare.14607741); i.e., changes in the composition of the prokaryotic communities were strongly associated with changes in the parasite communities.

**TABLE 1:** PERMANOVA and PERMDISP results^*a*^

Data set	Model structure	Variable	df	Pseudo-*F*	*R*^2^	*P*	*F*^*b*^
Prokaryotes	No strata (df_residual_ = 552)	Region	3	60.6	0.25	0.001	10.4***
		Sex	1	1.39	0.002	0.11	0.9
		Region × sex	3	1.15	0.005	0.19	NA

	Strata = region (df_residual_ = 502)	Habitat	3	28.33	0.1	0.001	52.4***
		Diet	8	15.07	0.13	0.001	16.1***
		Site	17	10.9	0.2	0.001	6.4***
		Sex	1	1.42	0.002	0.02	0.9
		Site × sex	28	0.98	0.03	0.68	NA

Parasites	No strata (df_residual_ = 552)	Region	3	84.96	0.31	0.001	5.0**
		Sex	1	2.02	0.002	0.07	0.6
		Region × sex	3	1.41	0.005	0.12	NA

	Strata = region (df_residual_ = 502)	Habitat	3	20.74	0.06	0.001	16.9***
		Diet	8	20.04	0.16	0.001	12.7***
		Site	17	13.98	0.24	0.001	2.0**
		Sex	1	2.21	0.002	0.04	0.6
		Site × sex	28	1.05	0.03	0.35	NA

a PERMANOVA and PERMDISP results showing degrees of freedom, pseudo-*F*, *R*^2^, and *P* values for PERMANOVA and *F* values with significance levels for PERMDISP for prokaryotes and parasites. For each data set, two models were run. The first model tested for the effect of region, sex, and their interaction, and the second model, stratified by region because of the large effect of region found in the first model, tested for effects of habitat, diet (consumption of algae, honey, nuts, termites, which typically require tools to access, see Table S1 at https://doi.org/10.6084/m9.figshare.14607687), site, sex, and a site-sex interaction. Note that the order of variable input matters but causes only minor changes in *R*^2^ values. For prokaryotes, if the order of habitat and diet is switched, habitat *R*^2^ = 0.08 and diet *R*^2^ = 0.15 (a change of 0.02). For parasites, if the order of habitat and diet is switched, habitat *R*^2^ = 0.08 and diet *R*^2^ = 0.15 (a change of 0.02 and 0.01).

b *F* values with significance levels for PERMDISP for prokaryotes and parasites are shown as follows: **, *P* < 0.01, ***, *P* < 0.001. NA, not available.

**FIG 4:**
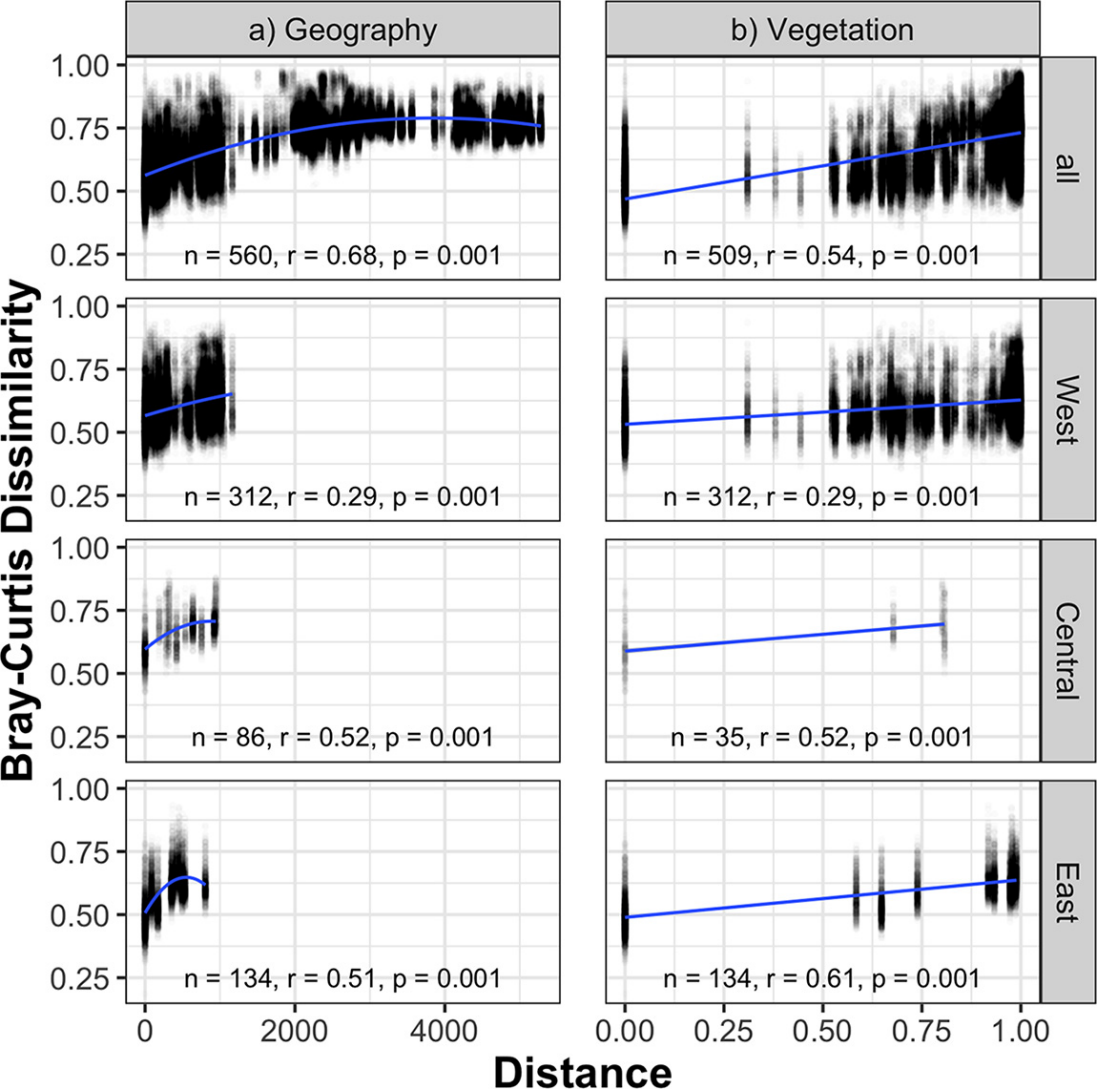
Prokaryote Bray-Curtis dissimilarity as a function of geographic distance (in kilometers), and vegetation dissimilarity (Bray-Curtis) for the whole data set and within the West, Central, and East regions. Within-region analyses for Nigeria-Cameroon are not shown here because there are only two sites. Statistics are from Mantel tests with Pearson *r* values. To aid in visualization, quadratic (geography) and linear (vegetation) models are displayed. To view similar figures in relation to climate distance, rather than geographic distance, see Fig. S2 at https://doi.org/10.6084/m9.figshare.14607735. To view similar figures in relation to genetic distance, see Fig. S3 at https://doi.org/10.6084/m9.figshare.14607738.

Across the whole data set, geographic, genetic, climate, and vegetation distances were all significantly correlated with prokaryotic community dissimilarity and parasite community dissimilarity (Mantel test, *P* = 0.001, Fig. 4; see also Fig. S2 [https://doi.org/10.6084/m9.figshare.14607735], Fig. S3 [https://doi.org/10.6084/m9.figshare.14607738], and Fig. S5 [https://doi.org/10.6084/m9.figshare.14607744]). The generalized dissimilarity model (GDM) with geographic and vegetation predictor matrices indicated that geographic distance explained the most variation in prokaryote and parasite communities across the whole data set (Table 2). For prokaryotes, partial Mantel tests of vegetation while controlling for geographic distance were significant (*r* = 0.27, *P* = 0.001). In other words, once we accounted for the conjoined effects of geography and genetics, vegetation still had a significant influence on prokaryotic community composition. For parasites, partial Mantel tests of vegetation while controlling for geography were also significant (*r* = 0.08, *P* = 0.001).

**TABLE 2:** Generalized dissimilarity modeling results^*a*^

Data set	Predictor matrix	All regions	West region	Central region	East region
Prokaryotes	Geography	**0.697**	0.097	0.133	0.000
	Vegetation	0.067	**0.174**	**0.213**	**0.344**
	% deviance explained	56.29	9.75	26.67	37.99

Parasites	Geography	**0.606**	0.057	0.000	**0.180**
	Vegetation	0.000	**0.111**	**0.265**	0.000
	% deviance explained	22.54	1.29	8.22	1.58

a Generalized dissimilarity modeling results showing the maximum partial ecological distance explained by geographic distance and vegetation dissimilarity predictor matrices while controlling for the other, as well as the deviance explained by the model. Models were run with the whole data set as well as within the West, Central, and East regions. Boldface values are the most important predictors in each data set.

At the prokaryote ASV level, there were 45, 7, 15, and 15 indicator ASVs for the West, Nigeria-Cameroon, Central, and East regions, respectively; indicator taxa for a region are both more abundant and more common in that region (48). These indicator taxa were randomly distributed across the phylogenetic tree (see Fig. S6a at https://doi.org/10.6084/m9.figshare.14607750), highlighting that they are not restricted to particular prokaryotic lineages and contain both spore-forming and non-spore-forming taxa.

### Variation in gut microbiota within regions.

To examine local effects and control for genetic differences, we also performed analyses within regions. Such analyses are also important to understand the extent to which broad patterns are generalizable or are contingent upon the strong differences observed across regions. Community dissimilarities of both prokaryotes and parasites were significantly greater in between-site sample comparisons of the entire data set than in within-site sample comparisons (Wilcoxon test, *P* < 0.001), and this pattern persisted in analyses within the four geographic regions (see Fig. S7 at https://doi.org/10.6084/m9.figshare.14607756). Within geographic regions, habitat type, diet (consumption of algae, honey, nuts, and termites, all of which are often consumed with tools), site identity, and to a lesser extent sex, affected prokaryotic and parasite community composition (Table 1). As with regional differences, there were strong differences in bacterial family abundances and parasite prevalence among sites in the same region (Fig. 2). Strikingly, certain parasite taxa had no occurrences in some sites and almost 100% prevalence at other sites within the same region (Fig. 2b).

Geographic, climate, and vegetation distances were all significantly correlated with prokaryotic community dissimilarity and parasite community dissimilarity within each region represented by more than two sites (i.e., three regions) (Mantel test, *P* = 0.001, Fig. 4; see also Fig. S2b [https://doi.org/10.6084/m9.figshare.14607735] and Fig. S5 [https://doi.org/10.6084/m9.figshare.14607744]). Individual-level genetic distances were significantly correlated with prokaryote community dissimilarity in all regions except for the West region and with parasite community dissimilarity in the Nigeria-Cameroon and Central regions (see Fig. S3 at http://doi.org/10.6084/m9.figshare.14607738). Additionally, parasite and prokaryotic community dissimilarity were significantly correlated within each region (Mantel test, *P* = 0.001, see Fig. S4 at http://doi.org/10.6084/m9.figshare.14607741). For prokaryotes, partial Mantel tests of vegetation while controlling for geography were significant within the East (*r* = 0.40, *P* = 0.001), Central (*r* = 0.29, *P* = 0.001), and West (*r* = 0.09, *P* = 0.001) regions. On the other hand, for parasites, partial Mantel tests of vegetation while controlling for geography were significant only in the Central (*r* = 0.32, *P* = 0.001) and West (*r* = 0.04, *P* = 0.03) regions. Full GDM models within each region indicate that the relative importance of vegetation was greater than geography, in contrast to the models built from the whole data set (Table 2).

Some site-specific differences were observed and were more strongly associated with prokaryote community composition than parasite community composition (Table 1; see also Fig. S8 at https://doi.org/10.6084/m9.figshare.14390369), although no such associations were found in the West region (subspecies *verus*). Knowing that some chimpanzee populations use tools to acquire certain specialty items in their diet, we categorized each site by the observed collection of specialty items they consume. For example, from long-term data, it is known that chimpanzees at Gashaka in the Nigerian-Cameroon range consume honey (49), but not termites (50). At Mount (Mt.) Cameroon, neither has been observed after 1 year of observation, and the possible difference in consumption of these items may contribute to the different microbial community composition between the two sites (see Fig. S8a and b at the figshare URL above). The five sites in the East region contained two different specialty diets, honey (Bwindi, Gishwati, and Nyungwe) and algae and termites (Issa), and a site (Budongo) where chimpanzees were never observed to consume any of those items (over 1 year of observations), although longer-term observations are needed as consumption of honey and termites was observed in a neighboring community of chimpanzees (51, 52); these three groupings were associated with distinct prokaryote communities (Fig. S8a at the figshare URL above). In the Central region, gut prokaryotic composition at the one site where only termite consumption was observed (Mts. de Cristal) was significantly different than at the two sites where only honey consumption was observed (Lopé and Conkouati, Fig. S8a at the figshare URL above). At the long-term field site of Goualougo, both termites and honey are known to be consumed (53), and at Loango, underground honey is consumed with tools (54), while termite consumption without tools has been observed. For consumption of honey and termites, which occurs in all four regions, there were 80 and 61 prokaryotic indicator taxa, respectively, distributed across the phylogenetic tree (see Fig. S6b at https://doi.org/10.6084/m9.figshare.14607750). Indicator taxa were shared among more than one region in only a few instances, likely reflective of the broader differences in community composition among the regions. Together, these results suggest that tool use may affect the composition of the gut microbiome by making certain food items available, but we recognize that tool use is difficult to disentangle from other factors that may also differ across chimpanzee populations, including differences in consumption of the various plant foods that make up the bulk of the chimpanzee diet or other prey items (16).

### Comparison with humans.

**FIG 5:**
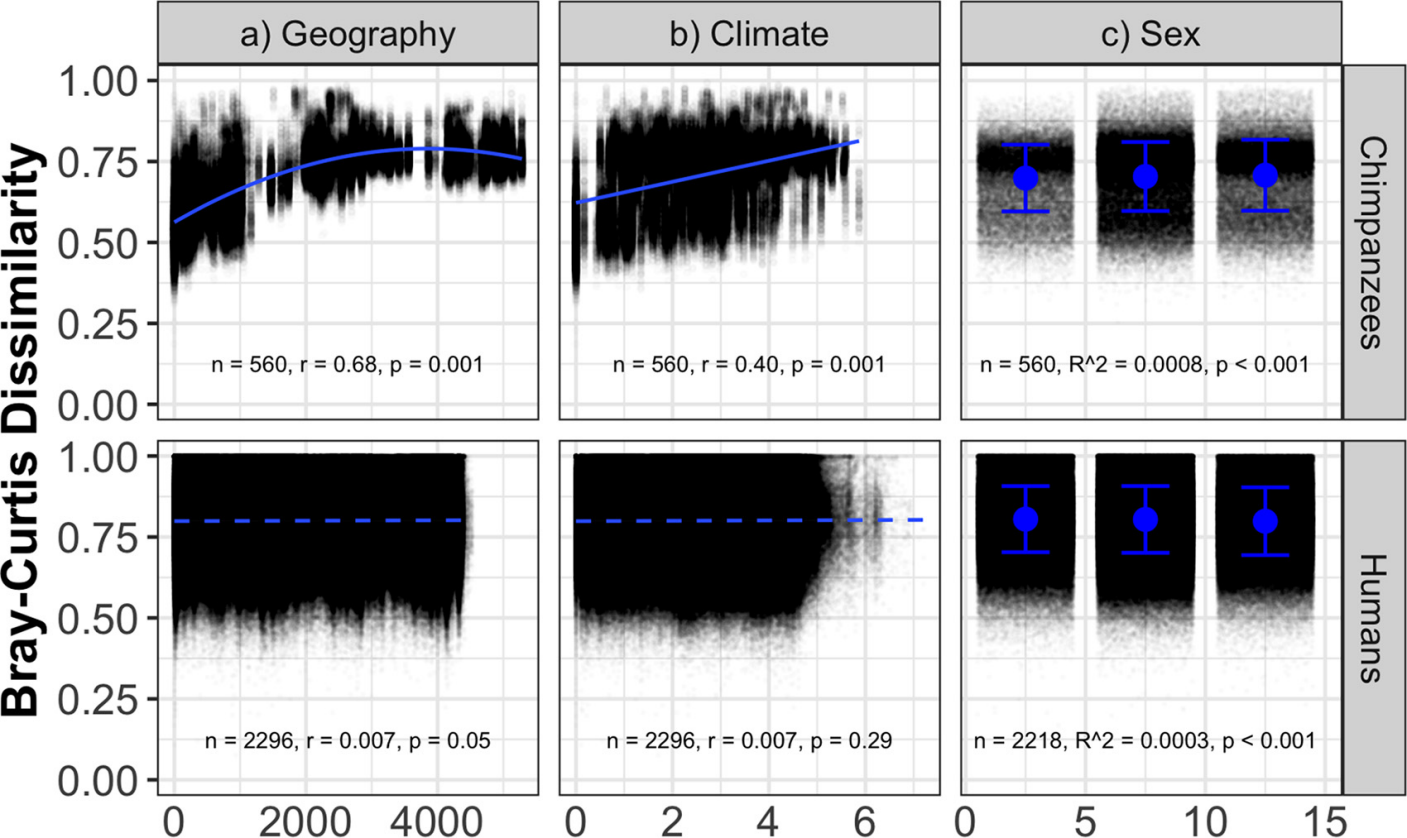
Relationship between geographic distance (in kilometers), climate distance (Euclidean distance), sex (FF = female-female comparisons, FM = female-male comparisons, MM = male-male comparisons), and prokaryote (bacteria and archaea) community composition in chimpanzees and humans. For chimpanzees, a quadratic (geography) and linear (climate) trendline is shown, while for humans a dashed, nonsignificant, linear trendline is shown. Blue points and error bars represent means and standard deviations, respectively. Statistics are from Mantel tests (geography and climate) and analysis of variance (ANOVA) (sex). Note that while there were significant effects of sex on dissimilarity, and male-male comparisons were more dissimilar than female-female and female-male comparisons in chimpanzees, but less dissimilar in humans, the extremely small effect sizes in both species suggests sex does not structure the gut microbiome in either species.

To contrast the effects of geography, climate, and sex on the gut microbiome in chimpanzees versus a population of industrialized humans known to have a relatively homogeneous diet and a high degree of interregional connectivity, we analyzed a comparable data set from humans enrolled in the American Gut Project (17) (see Materials and Methods). In contrast to the patterns observed for the chimpanzee gut microbiome, geographic distance and climate distance were not significantly correlated with human gut microbiome dissimilarity (Mantel test, *P* ≥ 0.05, Fig. 5a and b). While geography and climate had significant and strong (*r* = 0.66 and 0.44, respectively) effects on the chimpanzee gut microbiome, these same factors explained very little variation (*r* = 0.007) in the human gut microbiome. The effects of host sex were minor in both species (*R*^2^ = 0.0008 in chimpanzees, *R*^2^ = 0.0003 in humans, Fig. 5c).

## DISCUSSION

### Variation in gut microbiota among regions.

Previous studies on the chimpanzee gut microbiome have been limited in both sample size and geography, with most studies sampling only one of the four subspecies (e.g., references 50, 55, and 56). Our comprehensive sampling scheme enabled us to examine geographic and genetic effects on the chimpanzee microbiome. To date, few studies of any taxon have considered the variation in microbiomes among individuals within a species at such a scale, except for humans (57–59). On the basis of studies of humans (17, 60, 61), we expected there to be a weak effect of geographic and genetic distance on chimpanzee gut microbiota. Instead, we found that much of the variation among chimpanzees’ gut microbiomes was associated with geographic distance and genetic distance. This pattern was observed for both prokaryotes and eukaryotic parasites, including both parasitic worms and protists.

The effects of geographic and genetic distance were apparent both among and within geographic regions. Such effects could be due to either neutral divergence (42, 62, 63), disease (64), or some effect of host genetic identity on microbiome composition. The simplest (and most neutral) model is one in which microbiomes are inherited by individuals from their mother during birth and subsequently acquired from community members. The two predictions of this model, that geographic and genetic distance, which are strongly correlated in chimpanzees, are strongly predictive of prokaryote community composition and more weakly predictive of parasite community composition, are both supported by our data. The weaker predictive power for parasites is possibly because many parasite taxa (especially macroparasites) are acquired from dietary sources and the environment, in addition to directly from other chimpanzees via fecal-oral transmission (65), but it could also be due to the much lower number of parasite taxa compared to bacteria. Thus, we suspect that the relationship between host genetic differences and microbiome genetic differences primarily reflects history and neutral divergence. The relationship between genetic relatedness and gut microbiota observed at the continental scale in our data has not been previously documented, as previous work has focused on individual chimpanzee communities. Within individual chimpanzee communities, gut microbiome composition does not relate to genetic relatedness (e.g., parents and children and siblings) (66) but is rather influenced by social contact (67), with similar patterns also observed in baboons (5, 68), black howler monkeys (69), and sifakas (70). We cannot exclude the possibility of associations between specific host genes and microbiome composition, but we could not identify such associations here as the microsatellite genes provide information only on host genetic divergence.

Alternatively, some of the observed effects of geography and host genetics may be attributed to climate and vegetation, which were both correlated with geography (see Fig. S2 at https://doi.org/10.6084/m9.figshare.14607735), as well as tool use for special dietary items (e.g., hard-shell drupes are eaten only in part of the West region, see Table S1 at https://doi.org/10.6084/m9.figshare.14607687). Climate is unlikely to directly impact gut prokaryotes; many gut-associated prokaryotes cannot proliferate outside the gut. However, climate could affect eukaryotic members of the gut via effects on the presence and identity of alternate hosts at a particular site (65). In addition, climate can indirectly affect both prokaryotes and eukaryotes by influencing the vegetation composition and, consequently, the foods available at a particular site (71). Here, we find that across the entire chimpanzee range, the effects of climate, habitat type, and vegetation composition on the gut microbiome are modest, suggesting that the broad-scale geographic patterns we observed are only partially explained by climate and habitat.

In contrast to the pattern we observed for chimpanzees across sub-Saharan Africa, we found no geographic structure in the microbiomes of humans living across a similar geographic extent, the contiguous United States. Based on an analysis of 2296 samples, the microbiomes of humans in the contiguous United States are not correlated with geography or climate. For example, people in the United States living in New York and California can have as similar gut microbiota as two New Yorkers despite living in different climates 4,000 km apart. While other studies have shown differences in the human gut microbiome based on geography (e.g., reference 72), those studies reflect the effects of diet and lifestyle rather than geographic distance (18). Assuming that the patterns in gut microbiomes of chimpanzees are reflective of those of our last common ancestor, this lack of pattern in human microbiome data, at least in an industrialized Western society, suggests that humans have obscured ancient patterns in microbiome biogeography since diverging from chimpanzees, as has been observed with human effects on other ecosystems (56). This is likely due to a variety of processes associated with industrialization and globalization including, but not limited to, the decoupling of food and climate, and increased cross-regional traveling, movement, and social contact.

### Variation in gut microbiota within regions.

In addition to the broad regional differences we observed in chimpanzee microbiomes, there were also strong site-level differences within regions, which is likely amplified by higher rates of social contact among chimpanzees living in the same site (5, 67). Variation among sites within regions was correlated with geographic distance and genetic distance (just as with the among-region comparisons) but also with vegetation and consumption of specific diet items (16). It has been noted previously that even neighboring chimpanzee communities can have very different gut microbiomes (66). Here, we suggest that while some of the site differences are environmentally driven, the socially mediated use of tools may also possibly contribute to gut microbiome variation via diet.

Chimpanzees are one of the few species that employ the socially mediated use of tools, arguably a component of their foraging cultures (29, 31, 34, 38, 73), which influences which foods they eat and which tools they use to eat those foods. Consequently, differences between the foods available in a particular region and the foods actually consumed in a region may vary. The effect we observe of the consumption of algae, honey, nuts, and termites, which are consumed with the help of tools in many cases, suggests the potential influence of tool use on the gut microbiome via diet (44, 74).

Additional work is required to identify potential mechanisms of any associations between tool use and diet on microbiomes and the potential implications for host health. Here, we focused on four dietary items observed to be accessed using tools, although only one of them—nuts—is exclusively consumed when tools are used. Consumption of these four dietary items was possibly associated with differences in microbiome composition among sites in some instances. Since most of these items are eaten in large quantities, at least seasonally, it is possible that they offer different nutritional profiles that could select for different prokaryotic taxa in the gut. Algae, which are consumed at an average rate of ∼360 g/day during some months, are an important source of nutrients for chimpanzees (30) and feature recalcitrant complex carbohydrates; in fact, the gut bacteria of some human populations appear to have acquired the ability to produce specialized enzymes to digest seaweeds (75). Alternatively, honey provides high concentrations of simple sugars (76). In humans, sugar-rich diets are associated with increases in *Bifidobacteria* and decreases in *Bacteroides* (77); we observed one *Bifidobacteria* ASV associated with honey consumption in the Nigeria-Cameroon region, but this comparison should be made cautiously, as chimpanzee honey consumption is lower than human sugar consumption. The nuts such as oil-palm (Elaeis guineensis) and Coula edulis consumed by chimpanzees in the West region are rich in fat (78) and are consumed in large quantities in some months (700 g/day, net gain of 3,450 kcal/day) (72). Lastly, termites are sources of protein, iron, and manganese and are a valuable part of chimpanzee nutrition (79). However, we do not have comprehensive data on everything consumed by the chimpanzees at each site, and thus, there remain potential alternative explanations for site differences. For example, the three sites in the Central region where both honey and termites are consumed (Mts. de Cristal, Goualougo, and Loango) had distinct microbiomes, possibly due to the consumption of unique prey species, such as tortoises at Loango (80), or consumption of different fruit species. Furthermore, any differences between the Taï sites and other sites in the West region could be partially due to hunting of red colobus monkeys (16). While we do not have data on the frequency of tool use or the quantity of consumption for all of these specific diet items, we speculate that consumption of these items, if it is indeed contributing to these patterns, must be frequent and long term, as has been shown in certain sites (30, 76, 78, 79). For example, long-term dietary patterns in humans have far larger effects on the gut microbiome than short-term changes in diet (81). Continued long-term observations as well as DNA examination of feces for plants, arthropods, and other dietary items will provide additional insights into the relationships between diet and microbiome composition.

### Parasites.

There is a long history of research on parasites in primates (65), including many studies focused on chimpanzees (e.g., references 51 and 82). Until recently, however, parasite surveys used morphology-based identification, and newer sequencing studies have been limited in their geographic breadth or sample size (83–86). As a result, while we have a rich understanding of the presence of certain parasites in certain chimpanzee populations (65), our understanding of the distribution of parasites lags behind that of bacteria (85, 87). Our survey of gut microbial eukaryotic parasites sheds new light on parasite distributions in chimpanzees and the factors influencing those distributions. Only two parasites—one *Tetratrichomonas* ASV and the ciliate *Troglodytella abrassarti*—were present in all sites. *Troglodytella abrassarti* was not only found in all chimpanzee sites and in 94% of samples, but is also known to be common in other primates (88). This ciliate appears to be an important and beneficial component of the gut microbiome, contributing to polysaccharide hydrolytic activities in the chimpanzee colon (89). In contrast, most parasite species had more restricted patterns of distribution. Such between-site differences may be amplified by minimal social contact between sites, which is restricted to some immigration, while prevalence may be high within sites due to the social contact among individuals living at a site (87). In other primates, social contact has been observed to affect transmission of the nematode *Strongyloides* (82), but not protists (90, 91). Most parasites were also not consistently found in individuals with multiple samples, suggesting that they are transient in the gut and/or sourced from the environment or diet, or difficult to detect with 18S rRNA gene data (see Fig. S9 at https://doi.org/10.6084/m9.figshare.14390372).

As with the gut prokaryotes, dissimilarity in parasite communities was correlated with geography, climate, and vegetation, although the relationships for parasites were weaker than for prokaryotes, as has been found in other studies (85). Previous work has found more closely related primates to have more similar parasite communities (86), an observation we extend to the subspecies and population level. For example, parasites in the parabasalid *Tetratrichomonas* genus are prevalent in sympatric chimpanzees, gorillas, and humans, but different species and strains are found among the three species (84); different lineages within the genus are associated with different hosts, with a high degree of specificity (92). We report host specificity, at the regional level, in four of five trichomonads. Chimpanzees that eat insects as well as plants are expected to acquire some of the parasites present in the insects (65, 93), which likely contributed to the relationship between diet (e.g., termite consumption) and parasite community composition. Furthermore, the four items studied here contribute to chimpanzee nutrition, which may also influence parasite loads, as has been found in deer (94), bovids (95), and rats (96).

Dissimilarity among sites in prokaryotic communities and parasite communities was significantly correlated, which may be due to the fact that both prokaryotic communities and parasites are similarly influenced, both directly and indirectly, by geography, climate, and vegetation. It is also possible that the presence or absence of certain parasites can influence bacterial community composition or that the bacterial community influences parasite establishment. Many such transdomain correlations have been observed previously in both humans and chimpanzees (83, 97). In humans, the well-studied protozoan parasite *Blastocystis* is associated with lower abundances of *Bacteroides* bacteria (98), while the presence of *Entamoeba* can be predicted with 79% accuracy based on gut bacterial community composition (97). In chimpanzees, *Blastocystis* and *Strongyloides* carriers were associated with different bacterial communities (83). Several mechanisms of parasite-bacterium interactions have been proposed in the vertebrate gut (99). For example, parasites, including helminths and *Entamoeba*, can stimulate mucus production and alter mucosal composition, which may in turn affect nutrient availability and movement and attachment sites in the gut. Microbial eukaryotes can also play roles in nutrient cycling and bacterial turnover, and some, including *Entamoeba*, directly feed on bacteria (98, 100).

Many of the most prevalent parasites observed in our data set are common in humans and other mammals (88) and are associated with host health. *Dientamoeba*, *Blastocystis*, and *Entamoeba* are very common in humans and have been found to be more prevalent in healthy humans relative to humans with irritable bowel syndrome, inflammatory bowel disease, or other gastrointestinal disorders (98). Thus, these parasites may be potentially beneficial to the host under certain conditions (101). Alternatively, their prevalence may simply be a function of higher intestinal oxygen concentrations in humans with intestinal dysbiosis-linked diseases (98). In most cases, gut microbial eukaryotes move along the parasitism-mutualism spectrum in a context-dependent manner which may also be modulated by gut bacteria (99, 102).

### Conclusions.

Our geographic range-wide survey of the chimpanzee gut microbiome, including both prokaryotes and eukaryotic parasites, combining data on chimpanzee genetics, geography, climate, vegetation, and tool use to acquire specific foods, partially disentangles the drivers of gut microbiota composition across scales and synthesizes results from a number of previous local studies. The use of tool behavioral data in chimpanzees to explain differences in gut microbiota is a novel approach which may shed light on how diet, and the potential cultural behaviors that affect diet, can shape the gut microbiome and may have played a role in the evolution of the human microbiome. While isolation, either through genetic differences or geographic separation, outweighs climate, vegetation, and diet factors at large scales, climate, vegetation, and tool use all are correlated with gut microbiome composition at local scales, likely due to changes in diet. Geographic distance and climate played comparatively stronger roles in structuring the chimpanzee gut microbiome than the industrialized human gut microbiome, a product of technologies and developments that have decoupled humans from their local environments and food sources.

## MATERIALS AND METHODS

### Experimental design.

We collected feces from 29 different sites from a total of 14 countries spanning four main geographic regions of chimpanzee (Pan troglodytes) populations across the African continent (Fig. 1). To compile such a comprehensive sample set, samples were collected from multiple years and in all 12 months of the year, but year and season (wet/dry) had minimal effects on community composition (PERMANOVA *R*^2^ = 0.02 and 0.01, respectively, for prokaryotes and 0.02 and 0.02, respectively, for parasites). Regions are defined as West, Nigeria-Cameroon, Central, and East, which correspond to the Pan troglodytes *verus*, *ellioti*, *troglodytes*, and *schweinfurthii* subspecies, respectively. At each site, we aimed to collect feces from a single chimpanzee community, but because some of the groups are not habituated to human presence and therefore not observed directly, it is possible that samples from neighboring communities were inadvertently collected at a given site. Of the 560 samples used in the microbiome analysis, the number of samples per site ranged from 4 to 38 (see Table S2 at https://doi.org/10.6084/m9.figshare.14390420). Annual temperature, temperature seasonality (standard deviation × 100), annual precipitation, and precipitation seasonality (coefficient of variation) were extracted for each site from the BioClim data set (https://gitlab.com/tpoisot/BioClim/tree/master/assets). Vegetation composition data were collected according to the PanAf protocol (103) except for five sites which are described in Appendix 1 at https://doi.org/10.6084/m9.figshare.14390378. To summarize, at each site, transects spaced 1 km apart were established. Along these transects, 20 m × 20 m habitat plots were placed generally every 100 m (but up to 1,000 m); additional habitat plots were surveyed along gallery forest in sites with dominant savanna vegetation. The surveyed area ranged from 3 ha to 24 ha depending on the size of the site. Within each plot, all trees, lianas, and shrubs with a diameter at breast height (DBH) of ≥10 cm were taxonomically identified, and their diameter was measured at 1.3-m height. Site habitat type was defined broadly as either forest (mean ± SE plant species richness, 100 ± 11), forest mosaic (98 ± 21), savanna mosaic (78 ± 10), or savanna (58 ± 0) following previous work (104). Data on tool use behaviors to acquire algae, honey, nuts (technically hard-shell drupes), and termites come from the following sources: (i) extensive camera trap footage; (ii) fecal samples that provided evidence of ingestion of insects, algae, and honey, resources often exploited with the aid of tools; and (iii) evidence of tool use identified during reconnaissance, line, and strip transect surveys (39, 105) (see Table S1 at https://doi.org/10.6084/m9.figshare.14607687). We selected these four food acquisition behaviors because they generally occur at fixed locations with artifacts and are therefore relatively easy to detect via camera traps, exhibit variation across communities rather than being universal traits of chimpanzees, and target unique dietary items that could potentially affect the gut microbiome even if the majority of the chimpanzee diet is fruit. There is evidence of tool use to eat algae (6 of 7 sites where algae is consumed), honey (10 of 13 sites), nuts (5 of 5 sites), and termites (9 of 12 sites); for the remaining sites, we do not know whether tools for these dietary items were used or not, only that it has not been documented thus far. We did not include analysis of data on the consumption of other items such as meat and bone marrow as it is beyond the scope of the current observational record to reliably distinguish presence versus true absence at a site. Thus, we recognize that we account for only a fraction of the potential dietary differences in chimpanzees.

### Feces collection, extraction, and individual identification.

Chimpanzee fecal samples were collected throughout the year and preserved according to the two-step ethanol-silica method (106), stored in the field for up to 2 years, and then stored in the lab at −20°C prior to extraction. Feces were up to 3 days old as determined by field staff. The effects of time between defecation and sampling have been shown to be minor (7). Due to the presence of dung beetles, rain, and maggots, ape feces generally do not persist for more than a few days (107). Furthermore, samples older than 3 days are highly degraded and generally do not yield viable DNA for reliable chimpanzee genotyping (108). DNA was extracted from the samples using the QIAamp 96 PowerFecal QIAcube HT robot and kit (Qiagen, Hilden, Germany) with a pretreatment step to improve DNA yield (40). Microsatellite genotypes were obtained for up to 15 microsatellite loci using a two-step multiplex PCR method (109) with slight modifications (40) and electrophoresing the amplicons using an ABI PRISM 3130 Genetic Analyser and GeneMapper v 3.7 software (Applied Biosystems, Foster City, CA, USA) to visualize and score the results manually. Individual identity was assigned based on matching genotypes at a minimum of seven loci (110) from at least three PCR replicates for homozygotes and at least two replicates for heterozygotes (40). A fixation index (Fst) genetic distance matrix at the site level was calculated from these genetic data. Additionally, an individual-level genetic distance matrix was calculated using the Smouse and Peakall metric (111) with the GenoDive software program (112).

### Microbiome analysis.

Extracted DNA was transported on dry ice to the Fierer lab at the University of Colorado Boulder for PCR amplification and sequencing using the primers and methods of the Earth Microbiome Project (113). For prokaryotes (bacteria and archaea), we targeted the V4 region of the 16S rRNA gene using the 515F/806R primer pair, modified to include the necessary Illumina adapters. For eukaryotes, we targeted the V9 region of the 18S rRNA gene using the 1391f/EukBr primer pair. Following PCR, DNA was pooled, normalized with the SequalPrep normalization plate kit (Invitrogen, Carlsbad, CA, USA), and then sequenced on the Illumina MiSeq platform using 2 × 150 bp chemistry at the BioFrontiers Institute (Boulder, CO, USA). Amplicon reads were demultiplexed using the open source “idemp” tool (https://github.com/yhwu/idemp), and adapters were cut from the sequences using the open source “cutadapt” tool (114) (https://cutadapt.readthedocs.io/en/stable/) with default parameters and --minimum-length set at 50. Sequences were then quality filtered (16S parameters maxEE = 1, truncQ = 11, maxN = 0; 18S parameters maxEE = 2, truncQ = 2, maxN = 0), trimmed (150 and 145 bp for 16S, 103 bp for 18S) and merged (only 16S) using the DADA2 pipeline (115) to then infer amplicon sequence variants (ASVs) (116) and remove chimeras. 18S rRNA gene reads were not merged, and only the forward reads were used due to the variable length of the amplified region. Using the DADA2 pipeline, taxonomy was assigned using the SILVA database (117) version 132 (https://www.arb-silva.de/) for 16S rRNA sequences and the PR2 database (118) (https://pr2-database.org/) for 18S rRNA sequences. A phylogenetic tree of the 278 most abundant (>0.0625% mean relative abundance) and ubiquitous (present in >5% of samples) bacteria and archaea was constructed by using PyNAST (119) to align sequences and FastTree (120) to construct the tree using the QIIME program (121). Trees were visualized using the *ggtree* (122) R package. Eukaryote, chloroplast, and mitochondrial sequences were removed from the 16S rRNA sequence data set, as well as any ASV not assigned to either the bacterial or archaeal domains. 16S rRNA gene sequence data were then rarefied to 8,000 sequences per sample. Taxonomic filtering and rarefaction were performed using the *mctoolsr* (123) R package. This sequencing depth is adequate to capture most of the richness of ASVs in each sample, which ranged from an average of 196 to 247 ASVs per sample depending on the region (see Fig. S10 at https://doi.org/10.6084/m9.figshare.14390375). 18S rRNA gene sequence data were not rarefied, but samples with less than 3,100 sequences per sample before any taxonomic filtering were removed (samples should also have abundant plant and chimpanzee DNA). We identified 11 different ASVs that were present in at least 10% of samples, all of which were from known parasite species based on the literature (134). We took a conservative approach (to avoid false-positive results) and defined presence as having ≥50 sequences in a sample. To avoid counting two ASVs that likely represent the same parasite taxon as separate taxa, we ran correlations on read abundances and used BLAST analysis of ASV sequences for any ASVs identified as belonging to the same family, combining those ASVs that were strongly correlated and identified as belonging to the same species. Two of the ASVs that were significantly and strongly correlated (*r* = 0.82, *P* < 0.001) and closely related (percent identity = 98.06) were combined. Furthermore, due to previous work (83, 124) on known chimpanzee parasites from the *Blastocystis*, *Strongyloides*, and *Entamoeba* genera, we included a *Blastocystis* ASV, a *Strongyloides* ASV, and two *Entamoeba* ASVs that were present in at least 5% of samples, for a total of 14 parasite ASVs (see Table S3 at https://doi.org/10.6084/m9.figshare.14390426). These most prevalent and known chimpanzee parasites are unlikely to be sourced from prey species (93).

### Statistical analysis.

A total of 852 fecal samples passed the rarefaction cutoff of 8,000 16S rRNA gene reads per sample. These samples originated from 577 unique individuals (based on the microsatellite analysis described above), 147 of which were sampled multiple times, and 17 of which did not have sex identification. Within the same site, prokaryotic community Bray-Curtis dissimilarity was significantly greater between individual chimpanzees than within the same individuals that were sampled multiple times (see Fig. S11 at https://doi.org/10.6084/m9.figshare.14390381). To avoid statistical issues due to repeated sampling, we included only one sample per individual (using the first sample of each individual) in the analysis. Thus, we focused analyses on 560 fecal samples that originated from unique individuals and for which sex was identified (257 female, 303 male). To assess differences in community composition, we calculated Bray-Curtis dissimilarity for the prokaryote data and Jaccard dissimilarity for parasites. We conducted Wilcoxon tests to analyze whether Bray-Curtis or Jaccard dissimilarities were significantly different within versus among sampling sites, both on the entire data set and within each geographic region. We conducted principal coordinates analysis to visualize differences among samples with regard to genetic distance, climate distance, vegetation distance, prokaryote community composition, and parasite community composition. We used permutational multivariate analysis of variance (125) (PERMANOVA) implemented in the *vegan* (126) R package to assess the effects of geographic region on genetic, climate, and vegetation distance and the effects of geographic region, habitat type, diet, site identity, and sex on prokaryotic and parasite community composition. Pairwise PERMANOVAs were performed with the *RVAideMemoire* (127) R package. Multivariate homogeneity of group dispersions was tested with permutational analysis of multivariate dispersions (PERMDISP) implemented in *vegan*. We used Kruskal-Wallis tests with Bonferroni *P* value correction to analyze differences in the mean relative abundances of top bacterial families among the four geographic regions and logistic regressions to analyze differences in parasite occurrence probabilities among the regions. We used multilevel pattern analysis implemented in the *indicspecies* (48) R package to identify indicator taxa for each geographic region as well as for the presence and absence of consumption of honey and termites, which are consumed in all four regions (algae are consumed in two regions and nuts in one region).

To analyze the effects of geographic distance, climate, and vegetation on prokaryote and parasite community composition, we used Mantel tests and partial Mantel tests (controlling for geographic distance) between distance/dissimilarity matrices. Additionally, to further assess the relative importance of these predictor matrices, we used generalized dissimilarity modeling (128) (GDM) implemented in the *gdm* (129) R package. These analyses were performed on the entire data set as well as separately within the West, Central, and East regions; within-region analyses were not performed for Nigeria-Cameroon as there were only two sampling sites in this region. Climate distance was calculated as the Euclidean distance of annual mean temperature, annual mean precipitation, temperature seasonality, and precipitation seasonality, which were scaled (to 0 mean unit variance) before the calculation. Vegetation “distance” was calculated as Bray-Curtis dissimilarity in plant species composition.

To contrast the effects of geography, climate, and sex on the gut microbiome in chimpanzees versus in Westernized humans, we conducted Mantel tests between geographic distance, climate distance, and prokaryotic community Bray-Curtis dissimilarity for 2,296 human gut samples from the American Gut Project (17). This data set has readily and publicly available ASV tables, a similar geographic extent and sample size as our study, and used the same primers as our study. As previous studies have already investigated differences between the microbiomes of nonhuman primates and humans in different locations and societies (19, 42, 63, 130, 131), the goal here was simply to contrast the effects of geographic distance, climate, and sex in chimpanzees with a population of industrialized humans known to have a relatively homogeneous diet and a high degree of interregional connectivity. The effect of sex was assessed by comparing Bray-Curtis dissimilarities among female-female, female-male, and male-male pairwise comparisons. The human samples are from adults in the contiguous United States who did not take antibiotics in the year prior to sampling. Samples were rarefied at 10,000 sequences per sample and processed using Deblur (132) as outlined in reference 17. Data are publicly available and were downloaded from ftp://ftp.microbio.me/AmericanGut/manuscript-package/10000/distance/ on 24 March 2020.

### Data availability.

Raw sequencing data and ASV sequences were deposited to NCBI SRA and GenBank, respectively, under the BioProject accession number PRJNA625726.

## Supplementary Material

Reviewer comments
